# Impact of intra-articular injection with tranexamic acid on total blood loss and postoperative pain after arthroscopic rotator cuff repair surgery

**DOI:** 10.3389/fsurg.2023.1052039

**Published:** 2023-02-23

**Authors:** Rangteng Zhu, Hantao Jiang, Wei Xu, Liping Shen, Gang Jin

**Affiliations:** ^1^ Department of Orthopedics, Taizhou Hospital of Zhejiang Province Affiliated to Wenzhou Medical University, Taizhou, China; ^2^ Department of Clinical Laboratory, Taizhou Hospital of Zhejiang Province Affiliated to Wenzhou Medical University, Taizhou, China

**Keywords:** rotator cuff tear, arthroscopic rotator cuff repair, tranexamic acid, blood loss, pain, retrospective study

## Abstract

**Objectives:**

To evaluate the impact of intra-articular injection with tranexamic acid (TXA) on total blood loss (TBL) and postoperative pain after arthroscopic rotator cuff repair (ARCR).

**Methods:**

This study retrospectively included patients with full-thickness rotator cuff tears who underwent shoulder ARCR surgery in Taizhou hospital, China, between January 2018 and December 2020. Patients received 10 ml (100 mg/ml) of intra-articular TXA injection (TXA group) or 10 ml of normal saline (non-TXA group) after the incision was sutured. The primary variable was the type of drug injected into the shoulder joint at the end of the operation. The primary outcome were perioperative TBL and postoperative pain [measured by visual analog scale (VAS)]. The secondary outcomes were differences in red blood cell count, hemoglobin count, hematocrit, platelet count.

**Results:**

A total of 162 patients were included, 83 patients in TXA group and 79 patients in non-TXA group. Notably, patients in TXA group were more likely to have lower TBL volume [261.21 (175.13–506.67) ml vs. 382.41 (236.11–593.31), *P* = 0.025], and postoperative VAS score ≤ 2 within 24 h (*P* = 0.031) compared with those in non-TXA group. In addition, the median hemoglobin count difference was significantly lower in TXA group than that of in non-TXA group (*P* = 0.045), while, the differences in median counts of red blood cell, hematocrit, and platelet between the two groups were comparable (all *P* > 0.05).

**Conclusion:**

Intra-articular injection of TXA might reduce the TBL and degree of postoperative pain within 24 h after shoulder arthroscopy.

## 1. Introduction

Rotator cuff tears (RCTs) are a common shoulder disease that leads to the long-term pain and stiffness of the shoulder joint (1), often requiring rotator cuff repair surgery to restore function and relieve pain in the affected shoulder (2). As concepts and techniques improve, the number of arthroscopic rotator cuff repair (ARCR) interventions is increasing (3). Based on a previous research, it has been long believed that ARCR leads to only minimal blood loss (4). However, recent study by Liu et al. (5) reported that on the first postoperative day the estimated blood loss was approximately 300 ml, and the mean serum hemoglobin decreased by approximately 10 g/L. Thus, the bleeding from the surgical site can still affect the well-being of the patients and should be further discussed.

Tranexamic acid (TXA) is a synthetic analog of the amino acid lysine that competitively blocks the plasminogen lysine binding site, thereby inhibiting fibrinolysis (6). In recent years, TXA has been increasingly used in arthroscopic surgery to reduce intraoperative and postoperative bleeding. Previous studies have shown that intravenous administration of TXA before arthroscopic shoulder surgery could significantly reduce intraoperative bleeding (5), and intra-articular injection of TXA could reduce postoperative intra-articular bleeding on the first postoperative day in patients receiving arthroscopic anterior cruciate ligament construction surgery (7). To the best of our knowledge, no studies on the intra-articular injection of TXA in reducing intra-articular bleeding after ARCR have been published.

This study aimed to evaluate the impact of intra-articular injection with TXA on total blood loss (TBL) and postoperative pain after arthroscopic rotator cuff repair.

## 2. Methods

### 2.1. Study design and patients

This retrospective study included patients with RCTs who underwent shoulder arthroscopic surgery in Taizhou hospital, China, between January 2018 and December 2020. Non-probability sampling was used for this study. This retrospective study included all eligible patients with RCTs who underwent shoulder arthroscopic surgery in Taizhou hospital, China, between January 2018 and December 2020. Intra-articular TXA has been routinely administrated since March 2019 (TXA group), while patients before March 2019 were only administered 10 ml of saline (non-TXA group). Therefore, the patients treated between January 2018 and February 2019 were included in the non-TXA group, and those treated from March 2019 to December 2020 were included in the TXA group. Approval of Taizhou hospital ethics committee was obtained before the commencement of this study (K20220713). The informed consent was waived by the ethics committee due to the retrospective nature of the study.

Included patients underwent ARCR after being diagnosed with full-thickness RCTs of the postero-superior cuff by MR examination. Patients with biceps tendon and subscapularis injury were also included in this study. All patients received conservative treatment for more than 3 months without symptom relief. The exclusion criteria were as follows: (1) labral pathology requiring repair; (2) acute traumatic rotator cuff tears; (3) anticoagulation therapy before surgery; (4) abnormal coagulation profile (prothrombin time or activated partial thromboplastin time) before surgery; (5) renal or liver disorders; (6) irreparable massive rotator cuff tear.

To our knowledge, no studies have been published providing recommendations on the optimal intra-articular TXA dosage specifically for shoulder arthroscopic surgery. However, Chiang et al. (7) used the same arthroscopic technique to reconstruct the anterior cruciate ligament. In this study, a 10 ml (concentration:100 mg/ml) of intra-articular injection of TXA was found to reduce postoperative intra-articular bleeding during the first 24 h post-surgery. In addition, it also reduced the pain and the hemarthrosis severity in the early postoperative period. No systemic side effects or toxicity requiring aspiration was noted during the follow-up period. Therefore, we decided to apply the same dosage of TXA in our study.

### 2.2. Sample size

The sample size was calculated based on the TBL in a pilot study. The pilot TXA group consisted of 10 patients randomly selected from the rotator cuff tears patients treated with intra-articular injection of TXA during shoulder arthroscopic surgery. The pilot non-TXA group consisted of 10 patients randomly selected from the rotator cuff tears patients with intra-articular injection of normal saline. Mean and standard deviation (SD) were calculated for each pilot group. The calculated effect size was estimated using an α equal of 0.05 and a power (1-β) of 0.8. The final calculation effect size was 0.48. Based on this calculation, we determined that at least 70 patients were required for each group to achieve statistically significant results. The G*Power software version 3.1.9.7 was used to calculate the sample size (available at http://www.gpower.hhu.de; Heinrich Heine-University of Dusseldorf, Dusseldorf, Germany).

### 2.3. Surgery procedure

All patients underwent the shoulder arthroscopic surgery with the same surgical technique and all surgeries were performed by the same surgeons. After receiving general anesthesia combined with interscalene plexus block, all patients were placed in the lateral decubitus position. Normal saline containing epinephrine (1 mg/3 L) was used for intra-articular irrigation. A standardized arthroscopic approach for surgery was used with all patients.

First, an arthroscopic intra-articular examination was performed in all cases to probe whether the patients had other intra-articular lesions. We used 1 to 2 suture anchors (4.5 mm, TWINFIX Ultra PK Suture Anchor, Smith & Nephew) to repair the subscapularis if the subscapularis tendon was torn and recored the type of subscapularis tendon tears. Meanwhile, we performed a biceps tenotomy if the patients had a superior labrum anterior and posterior (SLAP) lesion or degenerative biceps tendon.

Then, *via* subacromial space arthroscopic vision, we performed acromiolpasty in cases with subacromial impingement syndorme and recored the type of postero-superior cuff tears. The double-row suture-bridge technique was used to repair the cuff tears (4.5 mm, Healix Healix Anchor System, Depuy and 4.5 mm, TWINFIX Ultra PK Suture Anchor, Smith & Nephew). Irreparable type C4 postero-superior cuff tears were eliminated from this study.

At the end of the operation, using a 14G puncture needle, 10 ml of TXA (100 mg/ml) or normal saline was injected into the shoulder joint through a posterior approach.

### 2.4. Postoperative care

Postoperatively, the operative limb was protected with an abduction brace for at least six weeks. During the duration of the hospital stay, all patients received postoperative intravenous injection of fumiprofen (50 mg, twice daily, Beijing Tide, China) for pain control. Passive movements, such as passive abduction and pendulum movement, began on the first day after surgery. All patients were discharged on the third day after surgery. In the third month after surgery, sports and task-specific exercises were permitted. Full return to sports and overhead activities was not permitted until the sixth month after surgery.

### 2.5. Data collection and definition

The following clinical parameters were collected: gender, age, height, weight, body mass index (BMI), affected side, systolic blood pressure (SBP), diastolic blood pressure (DBP), arterial pressure, inducement (non, fall down, sports, accident), underlying diseases (hypertension, diabetes), red blood cell count difference, hemoglobin count difference, hematocrit difference, platelet count difference, and intraoperative variables including type of postero-superior cuff tears and subscapularis tears, number of patients underwent acromiolpasty or long head of biceps tendon (LHBT) tenotomy, and duration of the surgery.

Classification of postero-superior cuff tears (C1, C2, C3, C4) and subscapularis tears (type 1, type 2, type 3, type 4, type 5) were performed according to the International Society of Arthroscopy, Knee Surgery and Orthopaedic Sports Medicine (ISAKOS) classification system (8).

### 2.6. Outcomes

The primary outcomes were TBL and postoperative pain. The TBL was calculated using the following equation (9): TBL = 70 ml × Weight × 2 × (preoperative hematocrit—postoperative hematocrit)/(preoperative hematocrit + postoperative hematocrit). The postoperative pain was measured by visual analog scale (VAS) score. The secondary outcomes were differences in red blood cell count, hemoglobin count, hematocrit, platelet count.

### 2.7. Statistical analysis

All statistical analysis were performed by SPSS software (version 20.0, license number: 4B6MINO86Z4LZV9AA7GHEC89P5TRNTOHAA3XKX5YW7GM2SWHCCTAFYBL3B3IKPMM7I9N3MSTBXOO8VPKXZHSEXGST8; IBM Corp., Armonk, NY).

#### 2.7.1. Hypothese testing

We hypothesized that the intra-articular injection of TXA can reduce postoperative blood loss and pain. The blood loss was mainly evaluated by measuring the changes after surgery in TBL. The postoperative pain intensity was assessed using the VAS. The differences in the continuous variables between the 2 groups (TXA group and non-TXA group) were tested using the *t*-test for the normally distributed variables and the rank-sum test for the non-normally distributed variables. The null hypothesis tested is that the patients in TXA group and non-TXA group demonstrate the same postoperative blood loss and pain. The a level was set *a priori* at.05.

#### 2.7.2. Qualitative data

The qualitative data, including gender, affected side, inducement, underlying diseases, and the supraspinatus and subscapularis tendon tear type, the number of patients who underwent acromioplasty and LHBY tenotomy were presented as percentages and compared using the chi-squared (*χ*^2^) test. A *P*-value below 0.05 was considered statistically significant.

#### 2.7.3. Quantitative data

The quantitative data included age, height, weight, BMI, SBP, DBP, surgery duration, platelet count changes, and the VAS score. The normally distributed data were represented as mean ± standard deviation and compared using the *t*-test. Conversely, the non-normally distributed data were represented as median (quartile range) and compared using the rank-sum test. A *P*-value below 0.05 was considered statistically significant.

## 3. Results

A total of 162 patients were included, with 83 patients in the TXA group and 79 patients in non-TXA group. Patients in TXA group were 60.24 ± 9.27 years old, with BMI of 23.88 (22.23–25.26) kg/m^2^, and patients in and non-TXA groups were 60.34 ± 8.87 years, with BMI of 23.88 (22.41–26.03) kg/m^2^. The basic characteristics were comparable between patients in TXA group and non-TXA group (All *P* > 0.05) (Table 1). Additionally, the intraoperative variables including the type of postero-superior cuff and scapular tendon tears, number of patients underwent acromiolpasty or LHBT tenotomy, and the duration of the surgery were not significantly different between the two groups (All *P* > 0.05) (Table 1).

**Table 1:** Basic characteristics and variables during surgical procedure.

Variables	TXA (*n* = 83)	Non-TXA (*n* = 79)	*P* value
Sex, *n* (%)			0.09
Males	37 (44.6)	25 (31.6)	
Females	46 (55.4)	54 (68.4)	
Age, year	60.24 ± 9.27	60.34 ± 8.87	0.944
Height (cm)	161 (156–168)	160 (155–166)	0.287
Weight (mean, kg)	62.88 ± 8.31	63.31 ± 10.28	0.799
BMI (kg/m2)	23.88 (22.23–25.26)	23.88 (22.41–26.03)	0.500
Affected side, *n* (%)			0.291
Left	24 (28.9)	29 (36.7)	
Right	59 (71.1)	50 (63.3)	
SBP (mean, mmHg)	132.71 ± 13.80	129.61 ± 14.63	0.167
DBP (mean, mmHg)	81.15 ± 9.59	78.96 ± 9.11	0.139
Arterial pressure (mean, mmHg)	98.33 ± 10.02	95.84 ± 9.05	0.099
Inducement, *n* (%)			0.420
Non	61 (73.5)	58 (73.4)	
Fall down	13 (15.7)	16 (20.3)	
Sports	3 (3.6)	0 (0)	
Accident	6 (7.2)	5 (6.3)	
Underlying diseases, *n* (%)			
Hypertension	17 (20.5)	14 (17.7)	0.655
Diabetes	10 (12.0)	10 (12.7)	0.906
Type of postero-superior cuff tears, *n* (%)			0.341
C1	37 (44.6)	40 (50.6)	
C2	21 (25.3)	24 (30.4)	
C3	10 (12.0)	8 (10.1)	
C4	15 (18.1)	7 (8.9)	
Type of subscapularis tendon, n(%)			1
Type 1	8 (9.6)	5 (6.3)	
Type 2	5 (6.0)	2 (2.5)	
Type 3	0 (0)	0 (0)	
Type 4	2 (2.4)	2 (2.5)	
Type 5	0 (0)	0 (0)	
Acromiolpasty, *n* (%)	2 (2.4)	6 (7.6)	0.246
LHBT tenotomy, *n* (%)	10 (12.0)	6 (7.6)	0.342
Duration of the surgery	105 (90–120)	95 (85–110)	0.083

BMI, body mass index; SBP, systolic blood pressure; DBP, diastolic blood pressure; LHBT, long head of biceps tendon.

Notably, patients in TXA group were more likely to have postoperative VAS pain score ≤ 2 within 24 h (*P* = 0.031), but not 2 h or 48 h after surgery (Table 2). The TBL volume in TXA group was significantly lower than non-TXA group [261.21 (175.13–506.67) ml vs. 382.41 (236.11–593.31), *P* = 0.025]. The median hemoglobin count difference before and after surgery was also significantly lower in TXA group than that of in non-TXA group (*P* = 0.045), while, the differences in median counts of red blood cell, hematocrit, and platelet between the two groups were comparable (all *P* > 0.05) (Table 2).

**Table 2:** Outcomes.

Outcomes	TXA (*n* = 83)	Non-TXA (*n* = 79)	*P* value
TBL volume, ml	261.21 (175.13–506.67)	382.41 (236.11–593.31)	**0** **.** **025**
Pain score			
VAS (2 h)			0.83
1	8	8	
2	69	66	
3	6	5	
4	0	0	
VAS (24 h)			**0**.**031**
1	0	1	
2	66	49	
3	17	28	
4	0	1	
VAS (48 h)			0.310
1	9	13	
2	64	66	
3	6	4	
4	0	0	
Red blood cell count difference	0.37 (0.20–0.59)	0.42 (0.20–0.61)	0.429
Hemoglobin count difference	8 (5–16)	13 (7–17)	**0**.**045**
Hematocrit difference	0.024 (0.014–0.046)	0.04 (0.014–0.054)	0.255
Platelet count difference	18 (5–28)	24 (8–44)	0.059

TBL, total blood loss.

No thromboembolic adverse effects, wound complications, or infections were noted in either group at 3 months after surgery. At last follow-up visit, all patients were satisfied with the post-operative shoulder function *via* the effective rehabilitation. Regrettably, we do not have the detailed clinical outcome scores to correlate with the findings of our investigations.

## 4. Discussion

This study demonstrated that the TBL volume in patients who received intra-articular TXA injection during shoulder arthroscopy was significantly lower than in those who did not receive the injection. Patients in TXA group were also more likely to have postoperative VAS pain score ≤ 2 within 24 h than in the TXA group, indicating that TXA injection might reduce the TBL and degree of postoperative pain. The null hypothesis was rejected and the alternative hypothesis supported.

Controlling the intra-articular bleeding is important for recovery and prognosis after shoulder arthroscopy surgery. Previous studies have shown that TXA effectively decreased postoperative intra-articular bleeding in arthroscopic knee surgeries, such as partial meniscectomy (10) and anterior cruciate ligament reconstruction (11). However, prospective double-blind study by Li et al. (5) in 2020 reported that intravenous administration of TXA before shoulder arthroscopy significantly improved intra-operative bleeding, but not affected post-operative blood loss, red cell count, hemoglobin or hematocrit. In this study, intra-articular TXA injection during shoulder arthroscopy successfully prevented blood loss and hemoglobin loss—in line with the above knee surgery studies, but slightly different from Li study. To the best of our knowledge, there are no available clinical studies on the effect of intra-articular injection of TXA after ARCR, thus reported results are encouraging and worse to be further tested in the future prospective studies.

Hemarthrosis is considered to affect articular cartilage to a great degree, increasing the risk of postoperative infections, and leading to worse outcomes, especially in the early postoperative period (12, 13). Accordingly, reducing intra-articular bleeding is important to improve the joint mobility and decrease post-operative pain. Study by Nugent et al. (10) compared patients undergoing arthroscopic meniscectomy who received intravenous TXA and normal saline and concluded that TXA could improve early functional recovery and reduce postoperative hemarthrosis of the knee joint without increasing risk. In this study patients in TXA group were more likely to have postoperative VAS pain score ≤ 2 within 24 h after surgery, which was comparable with the results of TXA intravenous administration (6, 11) and intra-articular injection (7) in arthroscopic anterior cruciate ligament reconstruction, suggesting that the reduced amount of postoperative hemarthrosis could reduce the pain in the early postoperative period.

Currently, the drug delivery method for TXA in orthopedic surgical procedures remains under discussion. Although after intravenous administration TXA may efficiently penetrate large joints, it has a relatively short half-life (2 h) and might remain just above the effective plasma concentration for only 4 to 6 h (7). Several systematic meta-analyses have shown that topical (intra-articular) administration of TXA has comparable effectiveness to intravenous delivery in orthopedic surgical procedures (14–17). Moreover, some studies noted that intra-articular TXA administration may have the potential benefits of allowing higher local concentration while preventing the risk of adverse systemic effects (7, 18). Studies by Alshryda et al. (18) and Ahmed et al. (19) concluded that intra-articular TXA provides a greater benefit than intravenous in decreasing postoperative blood loss during arthroplasty. By binding to the lysine binding site of the plasminogen molecule, TXA could lessen the activation of plasminogen to plasmin, thus reducing the dissolution of the fibrin network and ultimately reducing the consumption of platelets needed for hemostasis (20). Therefore, based on the above and results of the present study, it could be suggested that local administration of TXA during shoulder arthroscopy exerts its effect in the early postoperative period without systemic risks.

Numerous studies evaluated the incidence of adverse events after the administration of TXA for arthroscopic surgery. A systematic review by Goldstein et al. found no incidence of deep vein thrombosis (DVT), infection, and arthrofibrosis events associated with the use of TXA perioperatively in arthroscopic surgery of anterior cruciate ligament reconstruction, meniscectomy, femoroacetabular impingement, and rotator curr repair (21, 22). In another systematic review by Sun et al., no wound infections or cardiac, thrombotic, or thromboembolic complications were recorded in patients treated with TXA for arthroscopic surgery (23). Similarly, in our study, no thromboembolic adverse effects, wound complications, or infections were noted in either group at 3 months follow-up. Although a longer follow-up period would have provided more information on the efficacy of TXA, many patients in our study did not attend follow-up appointments after 3 months. However, no thromboembolic adverse effects, wound complications, or infections were noted in the TXA and control groups. Based on these findings, we speculate that some patients may have missed the follow-up appointments because they did not experience any complications after surgery. Therefore based on our findings and those of previous studies, we concluded that intra-articular TXA is safe and effective at reducing postoperative bleeding and pain.

This study provided important clinical guidance of reducing postoperative intra-articular bleeding in patients who underwent ARCR surgery. Nevertheless, this study acknowledges the following limitations. Due to the retrospective nature, some selective bias might have been present. The optimal dosage, route, and timing of TXA application in ARCR surgery remains to be investigated. And, finally, longer follow-up is required to adequately detect adverse reactions to TXA. Further multicenter prospective studies with large sample size are needed to clarify these issues. Moreover, during the initial use of TXA, we only focused on the peri-operative total blood loss (TBL) and pain, as well as post-operative complications. We did not perform a detailed assessment of shoulder function during follow-up. Therefore, we were unable to evaluate the effect of intra-articular injection of TXA on the postoperative shoulder function of patients. This requires a more comprehensive and detailed test plan in the next prospective study.

In conclusion, this study suggested that intra-articular injection of TXA might reduce the TBL and degree of postoperative pain within 24 h after shoulder arthroscopy.

## Data Availability

The original contributions presented in the study are included in the article/Supplementary Material, further inquiries can be directed to the corresponding author/s.
